# Is heart rate variability a feasible method to determine anaerobic threshold in progressive resistance exercise in coronary artery disease?

**DOI:** 10.1590/bjpt-rbf.2014.0165

**Published:** 2016-06-16

**Authors:** Milena P. R. Sperling, Rodrigo P. Simões, Flávia C. R. Caruso, Renata G. Mendes, Ross Arena, Audrey Borghi-Silva

**Affiliations:** 1 Interunidades Bioengenharia (EESC/FMRP/IQSC), Universidade de São Paulo (USP), São Carlos, SP, Brazil; 2 Laboratório de Fisioterapia Cardiopulmonar, Departamento de Fisioterapia, Universidade Federal de São Carlos (UFSCar), São Carlos, SP, Brazil; 3 Integrative Physiology Laboratory, Department of Physical Therapy, College of Applied Health Sciences, University of Illinois at Chicago (UIC), Chicago, USA

**Keywords:** autonomic nervous system, anaerobic threshold, blood lactate, cardiac rehabilitation, cardiac disease, 1-RM test

## Abstract

**Background:**

Recent studies have shown that the magnitude of the metabolic and autonomic responses during progressive resistance exercise (PRE) is associated with the determination of the anaerobic threshold (AT). AT is an important parameter to determine intensity in dynamic exercise.

**Objectives:**

To investigate the metabolic and cardiac autonomic responses during dynamic resistance exercise in patients with Coronary Artery Disease (CAD).

**Method:**

Twenty men (age = 63±7 years) with CAD [Left Ventricular Ejection Fraction (LVEF) = 60±10%] underwent a PRE protocol on a leg press until maximal exertion. The protocol began at 10% of One Repetition Maximum Test (1-RM), with subsequent increases of 10% until maximal exhaustion. Heart Rate Variability (HRV) indices from Poincaré plots (SD1, SD2, SD1/SD2) and time domain (rMSSD and RMSM), and blood lactate were determined at rest and during PRE.

**Results:**

Significant alterations in HRV and blood lactate were observed starting at 30% of 1-RM (p<0.05). Bland-Altman plots revealed a consistent agreement between blood lactate threshold (LT) and rMSSD threshold (rMSSDT) and between LT and SD1 threshold (SD1T). Relative values of 1-RM in all LT, rMSSDT and SD1T did not differ (29%±5 vs 28%±5 vs 29%±5 Kg, respectively).

**Conclusion:**

HRV during PRE could be a feasible noninvasive method of determining AT in CAD patients to plan intensities during cardiac rehabilitation.

## BULLET POINTS

- Parasympathetic modulation was reduced during lower extremity resistance exercise.
- Anaerobic Threshold occurred at ≈30% of 1-RM in patients with CAD.
- HRV may prove to be a feasible tool in clinical practice to determine Anaerobic Threshold.
- HRV can be safe and appropriate method to determine exercise intensity in patients with CAD.

## Introduction

It is known that the combination of aerobic and resistance exercise for cardiac patients synergistically improves muscular strength and endurance, functional capacity, quality of life, cardiovascular function, metabolism, and cardiovascular risk profile^1^. In addition, resistance exercise is considered safe for both healthy elderly individuals and cardiac patients^1^ ^-^ ^4^.

The magnitude of the cardiovascular and ventilatory responses to exertional demands depends on the type of physical exercise and the intensity of effort^1^. With respect to exercise intensity, the anaerobic threshold (AT) is defined as a point above a given power value when the production of lactic acid is greater than the capacity for its utilization by body tissues^5^ ^-^ ^7^. The point past which blood lactate concentration increases progressively^5^ is an important parameter in determining submaximal exercise tolerance. The use of discontinuous protocols to assess functional capacity and determine AT are potentially advantageous as they reduce the inherent added risks incurred during maximum stress intensities^2^.

The ability of Heart Rate Variability (HRV) to determine changes in blood lactate and AT during resistance and aerobic exercise in healthy individuals has already been investigated^8^ ^,^ ^9^. Other studies have also examined the behavior of HRV indices during exercise in diabetic^10^, heart failure^11^, and elderly^12^ ^-^ ^14^ cohorts. However, parameters that indicate safe training intensities with resistance exercise, particularly in patients with cardiac conditions, remain unclear.

While HRV indices are important predictors of cardiovascular risk and risk of sudden cardiac death and may be used as potential indices of relative risk^15^, the use of HRV to determine the point of transition between aerobic and anaerobic metabolism (i.e., AT) during incremental resistance exercise in patients with cardiac disease is unknown. Therefore, the objectives of this study were to: 1) evaluate the behavior of HRV and blood lactate; 2) determine the AT during an incremental leg-press protocol with an incremental percentage of One Repetition Maximum Test (1-RM); and 3) evaluate the degree of agreement between HRV indices and blood lactate in relation to the AT in a cohort diagnosed with coronary artery disease (CAD).

## Method

### Study design and population

This is an observational cross-sectional study involving 20 males with clinically stable CAD (sample of convenience) participating in an outpatient cardiac rehabilitation program. Inclusion criteria consisted of 1) being at least 12 months post an acute event (i.e., myocardial infarction) or 12 months after a surgical or percutaneous revascularization procedure and 2) being clinically stable on a regular pharmacologic regimen.

The experimental protocol was approved by the Research Ethics Committee of Centro Universitário de Araraquara, Araraquara, SP, Brazil (n. 1331-11). All procedures were conducted in accordance with the Declaration of Helsinki. All participants signed an informed consent form.

### Experimental procedures

Subjects did not ingest caffeine or alcohol during the 24-hour period prior to any of the testing protocols and did not perform any rigorous physical activity during the 48 hours prior to testing. All trials were performed at the same period of the day to avoid any influence of circadian rhythm on cardiovascular variables. The experiments were carried out in a climate-controlled room (21-24 °C) with a relative air humidity of 40-60%.

Clinical examination was performed by a physician (cardiologist) before study initiation. This examination consisted of anamnesis and resting 12-lead electrocardiography. A transthoracic echocardiogram was also performed for all patients.

### Cardiopulmonary exercise testing – CPX

A symptom-limited incremental exercise test (CPX) was performed on a recumbent cycle ergometer (Corival, Lode, Groningen, The Netherlands) with the collection of gas exchange and ventilatory variables using a calibrated computer-based exercise system (Oxycon Mobile, Jaeger^TM^, Hoechberg, Germany).

The workload (W) was continuously increased in a linear “ramp” pattern of 15 W.min^–1^. The test finished when subjects reached physical exhaustion or when abnormal test responses warranted test termination^16^ ^,^ ^17^. The incremental exercise testing duration was between 8 and 12 minutes^18^.

Peak VO_2_ was defined as the highest value during the last 15 seconds of exercise and peak respiratory exchange ratio (RER) was the 15-second averaged VCO_2_ divided by VO_2_ at peak exercise^16^.

### One Repetition Maximum test – (1-RM - leg press)

This test was applied by gradually increasing the resistance until the patients succeeded in performing no more than one repetition on the leg press at a 45 degree angle (Pro-Fitness, São Paulo, Brazil). The resistance load for 1-RM was estimated (1-RM-E) before the test by multiplying subject body weight by 3.5, based on pilot testing. The details of this test protocol have been described previously^13^.

### Discontinuous resistance exercise testing (DRET-leg press)

72 hours after the 1-RM test, Discontinuous Resistance Exercise Testing (DRET–leg press) was performed, starting at a load of 10% of 1-RM with subsequent increases of 10% until exhaustion. At each percentage of effort, patients underwent 2 minutes of exercise at a movement rhythm of 12 repetitions/minute, maintaining respiratory cadence. The period between trials was 5 minutes. The details of this test protocol have been described previously^13^.

Heart Rate (HR) and R-R intervals were recorded with a wireless HR monitor (Polar S810i, Kempele, Finland) and blood samples (via earlobe puncture) were taken at rest and immediately after the final repetition completed at each load (i.e., % of 1-RM). Blood samples were analyzed using a YSI 1500 Sports Lactate Analyzer (YSI Inc., Yellow Springs, OH, USA).

### Measurement of HRV

The R-R intervals were recorded continuously with the wireless HR monitor (Polar S810i) during all exercise protocols. The R-Ri captured with the monitor can be analyzed with both linear and nonlinear models. After data capture, the signals were transmitted to a receiver and interface connected to a computer for subsequent analysis. The details of this technique have been described previously^13^.

### Safety during exercise protocols (CPX, 1-RM–leg press and DRET-leg press)

During all exercise protocols, HR was recorded with the HR monitor (Polar S810i) and the ECG was constantly monitored using a USB electrocardiogram (WinCardio, Micromed Biotecnologia, Brasilia, Brazil) to detect any potential arrhythmias or signs of ischemia that would indicate the protocol should be interrupted. Blood pressure (auscultation) and symptoms (muscle fatigue, chest pain, and breathing/dyspnea), assessed by means of the 10-point modified Borg Scale Rating^16^ ^,^ ^19^ ^-^ ^21^, were measured and recorded immediately after each effort.

The criteria for protocol termination were a systolic blood pressure >200 mmHg, symptoms of lower limb fatigue, angina or shortness of breath, development of any cardiac arrhythmias, or achieving maximum voluntary exhaustion^13^.

### Data analysis

To evaluate the responses of HR and R-Ri during DRET–leg press, the first step in the data analysis involved a visual inspection of R-Ri (ms) distribution in the ECG in order to select the sections corresponding to the final minute of each load (second minute) of resistance exercise maneuvers, as this was considered to be a more stable phase for analysis^22^ ^-^ ^24^.

Ectopic beats, arrhythmic events, missing data, and noise effects that might alter the estimation of HRV were excluded^15^. HRV analysis was carried out using the following linear and nonlinear methods: 1) Linear methods - RMSSD (square root of the mean of the sum of the squares of differences between adjacent RRi divided by the number of RRi minus one, expressed in ms) and RMSM (square root of the sum of the squares of differences of individual values compared to the mean value, divided by the number of RRi in a period for the time domain); and 2) nonlinear method - SD1 (instantaneous R-R interval variability from Poincaré plots), SD2 (standard deviation of continuous long-term R-R interval variability), and the SD1/SD2 ratio carried out by the Poincaré method of quantitative two-dimensional vector analysis^15^.

The Poincaré plots were analyzed quantitatively, based on the premise of different temporal effects of changes in vagal and sympathetic modulation of HR on the R-R intervals without a requirement for a stationary quality of the data^24^. RR-interval series were processed using Kubios HRV 2.0 (University of Kuopio, Finland). The details of this technique have been described previously^24^.

### Determination of Anaerobic Threshold (AT) in resistance exercise

To determine AT, changes in blood lactate curves were generated for each subject and the AT was defined as the exercise intensity at which the blood lactate concentration began to increase exponentially, i.e., breakpoint^8^ ^,^ ^12^ ^-^ ^14^.

To determine the HRV threshold, the rMSSD and SD1 for each stage of exercise were plotted against work rate. This HRV deflection point was defined as the HRV threshold^9^. The point at which there was an initial decline in indices during exercise, thus indicating vagal withdrawal.

The determination of the lactate and HRV threshold occurred through visual inspection of lactate and HRV curves, respectively, by two independent experienced examiners. When there was no agreement between the two evaluators, a third evaluator was called to give the casting vote.

### Statistical analysis

The sample size for the current study was estimated considering previous studies with the same resistance exercise protocol for healthy and elderly^13^ ^,^ ^14^ subjects and also different resistance exercise protocols for coronary artery disease^22^ ^,^ ^25^. Considering the presence of coronary artery disease, we doubled the sample size (n=20) to increase the chance of having less variability of the resulting data.

Initially, we used the Kolmogorov-Smirnov test to verify the normality of the data and subsequently one-way ANOVA with repeated measures was used to analyze the behavior of the HRV indices, blood lactate curves, R-Ri and RPP responses during the DRET–leg press (at different percentages of 1-RM), and the different methods of identifying AT (blood lactate curves, rMSSD, and SD1 threshold). When appropriate, post-hoc analyses were performed using the Tukey test. The degree of agreement between the methods used to determine AT was verified using Bland-Altman plots^26^ ^-^ ^28^.

Data are reported as mean and standard deviation and the significance level was set at 5%. The statistical analysis was carried out using Sigma Plot for Windows version 11.0 (Sigma Plot, San Jose, CA, USA) and MedCalc version 12.6.1.0 (MedCalc Software, Ostend, Belgium).

## Results

Over a one-year period, 42 patients were assessed for eligibility, 26 were recruited, five did not meet the inclusion criteria, and one was excluded for having an inadequate blood pressure response during CPX. Among the remaining subjects, 20 completed the protocol successfully with no abnormalities that would contraindicate enrollment in the present study and were included in the final analysis.

The clinical characteristics of the subjects are summarized in Table 1. All subjects had normal left ventricular systolic function (and mild left ventricle diastolic dysfunction in 45% of the study population) measured by echocardiography. The majority of patients had hypertension, history of smoking, and a family history of CAD. Myocardial infarction was the predominant clinical diagnosis and all patients were NYHA class I. Pharmacologic treatment commonly included antiplatelets, statins, beta-blockers, ACE inhibitors, and hypoglycemic agents. Mean CPX values indicate this sample had a well-preserved functional capacity and exerted maximal effort during the exercise test, according to American Heart Association (AHA) standards^16^.

**Table 1:** Baseline characteristics of the study population.

	CAD, n = 20
**Demographics/anthropometrics**	
Age, years	63±7
Height, m	1.7±0.1
Body mass, kg	75.7±12.7
BMI, kg/m^2^	26.6±2.9
**Transthoracic echocardiography**	
LVEF, %	60±10
LV diastolic diameter, cm	5.3±0.6
LV diastolic volume, ml	143±40
Septal thickness, cm	0.9±0.2
Posterior wall thickness, cm	0.9±0.1
**Doppler echocardiography**	
**LV diastolic function** ^*^ **:**	
Normal	11 (55)
Mild dysfunction	9 (45)
**Risk Factors, n (%)**	
Diabetes	5 (25)
Hypertension	13 (65)
History of smoking	12 (60)
Family history of CAD	18 (90)
**Functional Class (NYHA): I, n (%)**	20 (100)
**History of Myocardial infarction, n (%)**	15 (75)
**Intervention, n (%)**	
CABG	9 (45)
PCI	18 (90)
**Medications, n (%)**	
Antiplatelet (aspirin)	20 (100)
Statin	20 (100)
Beta-blocker	14 (70)
ACE inhibitor	7 (35)
Hypoglycemic	5 (25)
**CPX**	
Peak VO_2_, ml.Kg^–1^.min^–1^	24±5
PredictiveVO_2_, ml.Kg^–1^.min^–1^ (%)^*^	85±14
Peak workload, W	134±23

Data are presented as mean±SD or number (percentage) of subjects.

CAD: coronary artery disease; BMI: body mass index; NYHA: New York Heart Association; CABG: coronary artery bypass grafting; PCI: percutaneous coronary intervention; LVEF: left ventricular ejection fraction; ACE: angiotensin-converting enzyme; CPX: cardiopulmonary exercise testing; VO_2_: oxygen uptake; W: watts.

* Clinical recommendations for Cardiopulmonary Exercise Testing data assessment in specific patient populations^16^.

1-RM testing and DRET (30% and 50%) responses are summarized in Table 2. Regarding the response to the 1-RM testing, the criterion for termination for almost all subjects was muscle fatigue (rate of perceived exertion – RPE = 9.2±2.0), with only one test interrupted due to chest pain. No test was interrupted due to ECG alterations or an excessive rise in SBP (>200 mmHg). In relation to the resistance load achieved during 1-RM, values were similar to those stipulated previously during the pilot test (3.5 times the body weight of the patient). Regarding the response to the DRET, the criterion for termination for almost all subjects was muscle fatigue (RPE = 6.6±2.8) or an excessive rise in SBP (>200 mHg) and only one test was interrupted due to chest pain.

**Table 2:** Cardiovascular, metabolic, and cardiac autonomic variables obtained during peak 1-RM testing and DRET (30% and 50% of the 1-RM testing).

	CAD, n=20			
	**1-RM testing**	**DRET 30%**	**DRET 50%**	
**SBP**, mmHg	137±24	156±25	172±23	F=9.07
**DBP**, mmHg	76±14	87±11	89±18	F=4.47
**HR**, bpm	95±13	88±11	104a^±^16	F=6.15
**RPE**, 0-10 Lower limb fatigue Chest pain (angina) Breathing (dyspnea)	9.2±2.0 ^*^ 5.9±3.2	3.0±2.3 −− 2.8±2.4	6.6±2.8 ^*^ 5.9±2.9	F=35.79 F=9.22
**Load**, Kg	282±46	85±13c	144a,b^±^23	F=211
**Load 1RM/total body mass**	3.8±0.9	−−	−−	
**Lactate**, mlmol.L^–1^	−−	1.5±0.8	3.4±1.7a	F=28.60
**HRV indices** rMSSD RMSM SD1 SD2 SD1/SD2	−− −− −− −− −−	8.6±4.1 15.8±7.3 6.1±2.9 21.2±10.2 0.3±0.2	7.1±4.7 25.5a^±^16.0 5.1±3.3 35.5a^±^22.4 0.2±0.1	F=23.61 F=2.53 F=23.66 F=3.24 F=21.14

Data are presented as mean±SD.

SBP: systolic blood pressure; DBP: diastolic blood pressure; HR: heart rate; 1-RM: one repetition maximum; DRET: discontinuous resistance exercise testing; RPE: rate of perceived exertion.

* Only one patient had chest pain.

a: difference between DRET 50% and DRET 30%.

b: difference between DRET 50% and 1-RM testing.

c: difference between DRET 30% and 1-RM testing (p value <0.05, one-way ANOVA with repeated measures).

**Figure 1:**
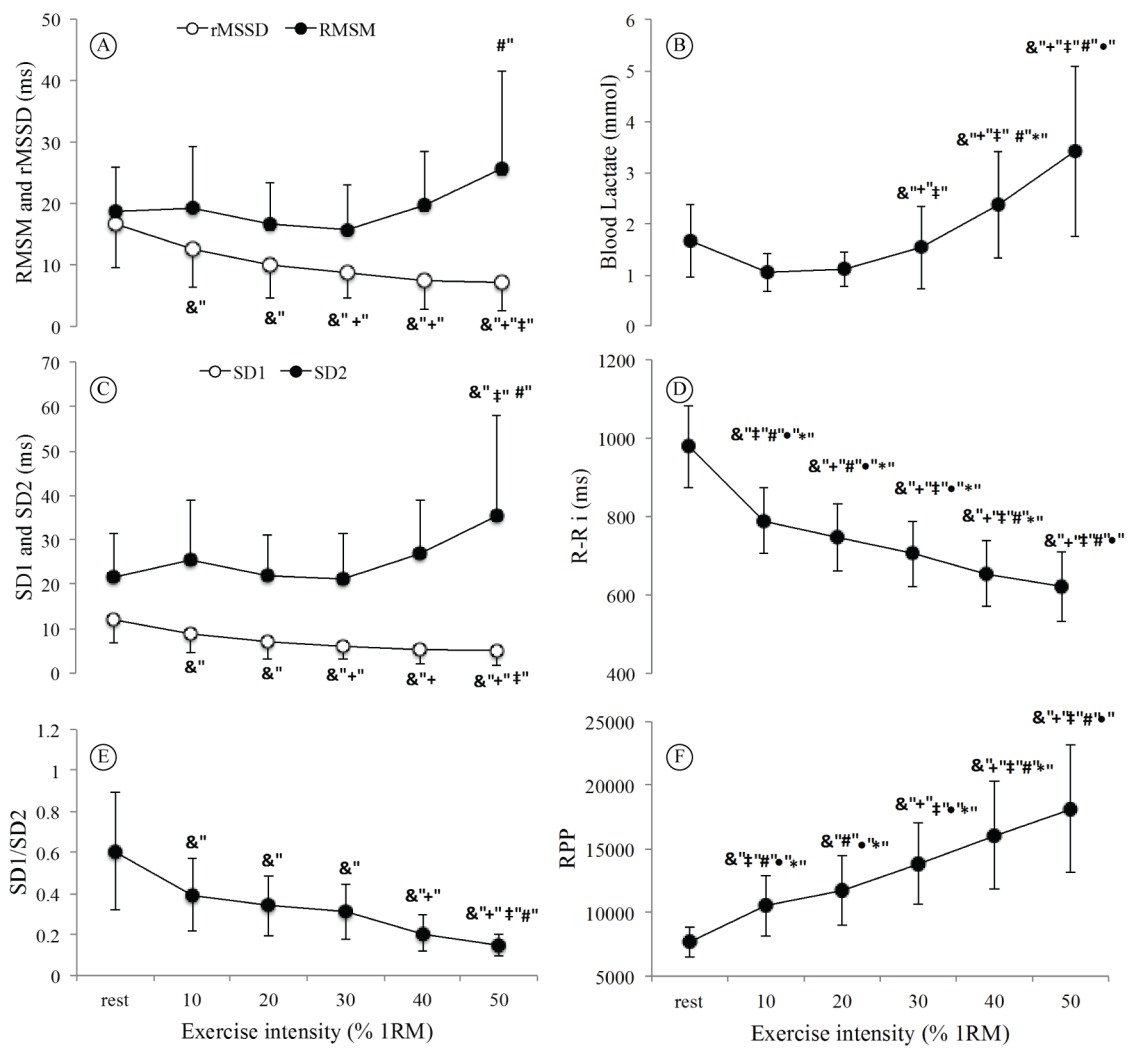
Behavior of variables in Discontinuous Resistance Exercise Testing (DRET) in percentage of 1 repetition maximum (1-RM; x axis), starting from rest until the load in common for all patients (50% of 1-RM). Data are presented as mean±SD. (A) rMSSD (square root of the difference in the sum of squares between R-R interval on the recording, divided by the determined time minus one) and RMSM (root mean square of the differences from the mean interval); (B) Blood Lactate; (C) SD1 (standard deviation of instantaneous beat-to-beat R-R interval variability) and SD2 (the standard deviation of continuous long-term R-R interval. &: difference in relation to rest. +: difference in relation to 10% of 1-RM. ‡: difference in relation to 20% of 1-RM. #: difference in relation to 30% of 1-RM. •: difference in relation to 40% of 1-RM. *: difference in relation to 50% of 1-RM (one-way ANOVA with repeated measures; p<0.05).

Figure 1 illustrates the behavior of HRV indices, blood lactate, and R-Ri and RPP at rest and with the increasing resistance exercise loads through the common maximum load achieved by all patients (i.e., 50% of 1-RM). Both rMSSD and SD1 indices, which are representative of parasympathetic modulation, demonstrated a significant decrease at peak load compared to resting values, with a significant drop at 30% of 1-RM (Figure 1A, C) with a parallel significant increase in blood lactate at 30% of 1-RM (Figure 1B).

The SD1/SD2 ratio had a significant decrease from 40% of 1-RM (Figure 1E). The RMSM and SD2 indices (Figure 1A, C) were significantly increased at 50% of 1-RM, although there was an increasing trend starting in 30% of 1-RM, perceived visually. R-Ri showed a progressive reduction (Figure 1D) and RPP showed a progressive increase concurrently (Figure 1F), reflecting the progressive increase in the intensity of effort at 50% of 1-RM.

The AT was determined for each patient through the analysis of blood lactate curves, rMSSD, and SD1, expressed in both absolute and relative values (Table 3). There were no significant differences in relation to different methods for identifying absolute values in Kg (lactate threshold - LT: 81 ± 19, rMSSD threshold - rMSSDT: 78 ± 14; SD1 threshold - SD1T: 79 ± 13) and relative AT values at ≈30% of 1-RM (29 ± 5; 28 ± 5; 29 ± 5; respectively), as presented in Table 3.

**Table 3:** Comparison of relative and absolute resistance values for anaerobic threshold measured with different methods of identification during discontinuous resistance exercise testing (DRET).

	**LT**	**rMSSDT**	**SD1T**	
**DRET**				
Absolute values, Kg	81±19	78±14	79±13	p=0.43; F=0.94
Relative values, %	29±5	28±5	29±5	p=0.52; F=0.76

Data are presented as mean±SD.

LT: Lactate threshold; rMSSDT: rMSSD threshold; SD1T: SD1 threshold. No significant differences among the three methods of identifying the anaerobic threshold (one-way ANOVA with repeated measures).

**Figure 2:**
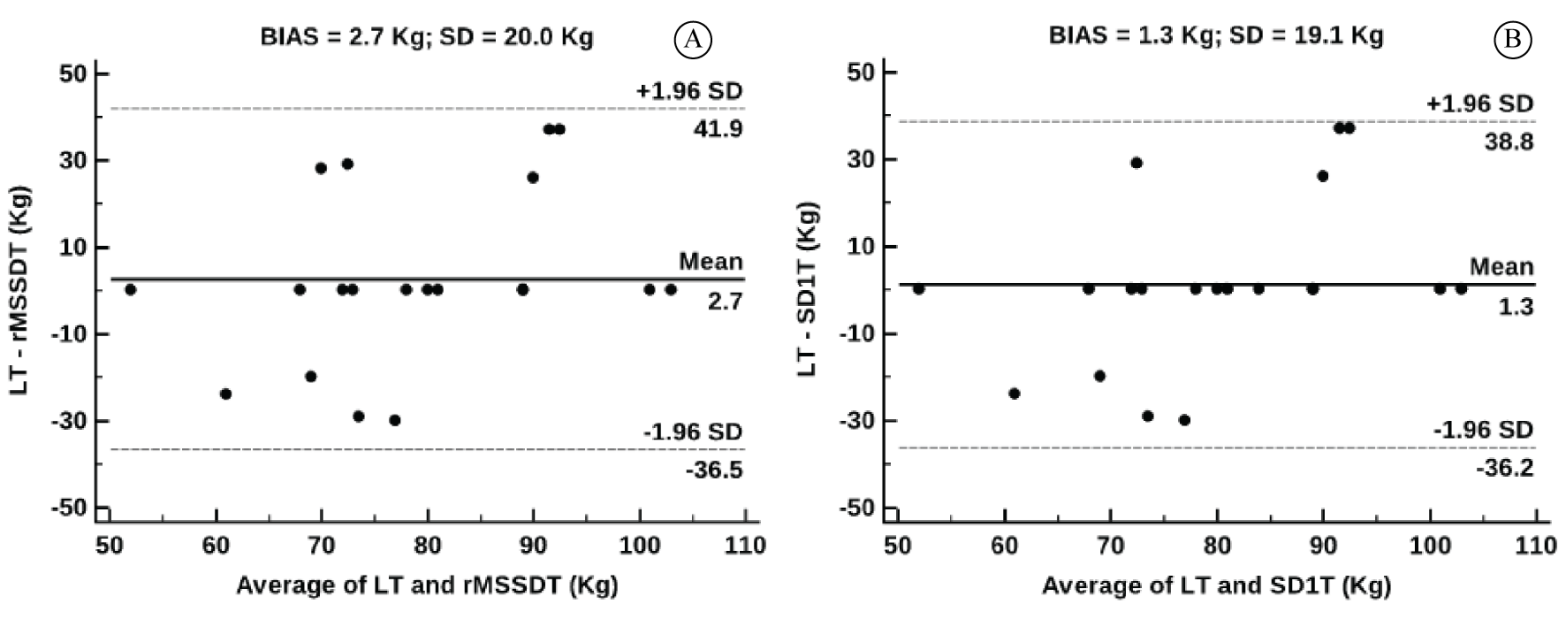
Bland-Altman plot showing the agreement between LT and rMSSDT (A) and LT and SD1T (B). BIAS = mean of the differences among the averages;±1.96 SD = 95% limits of agreement. LT = lactate threshold; rMSSDT = rMSSD threshold (rMSSD: square root of the mean of the sum of the squares of differences between adjacent RR-intervals on the recording, divided by the determined time minus one); SD1T = SD1 threshold (SD1: standard deviation of instantaneous R-R interval variability). Horizontal lines indicate mean (solid lines) and 95% confidence intervals (dashed lines) of differences between two measurements.

The analysis of agreement between methods of determining the AT was carried out using Bland-Altman plots, considering the blood lactate analyses as the “gold standard“. LT vs. rMSSDT and LT vs. SD1T were plotted. The mean of the differences for identifying AT using the LT and rMSSDT methods was 2.7 ± 20 Kg (Figure 2A), and the mean difference between LT and SD1T was 1.3 ± 19.1 Kg (Figure 2B), demonstrating good agreement.

## Discussion

In this observational cross-sectional study, we were able to demonstrate that the fall in parasympathetic indices is associated with an increase in blood lactate, starting at ≈30% of 1-RM using a leg-press maneuver. The good agreement between HRV indices and blood lactate curves may represent the importance and value of HRV in CAD patients for exercise prescription and monitoring.

### Responses during discontinuous resistance exercise

The rMSSD and SD1 indices reflect parasympathetic heart activity^15^ ^,^ ^24^ ^,^ ^28^ and they both demonstrated a significant drop from ≈30% of 1-RM (Figure 1A, C). The total HRV, represented by RMSM and SD2 indices (Figure 1A, C) were significantly increased from ≈50%of 1-RM, although the increasing trend started at ≈30% of 1-RM, observed visually. Lastly, the SD1/SD2 ratio appears stable up to ≈30% of 1-RM, followed by changes thereafter (Figure 1E). All of these changes indicate a shift in sympathovagal balance towards sympathetic predominance and reduced vagal tone^29^ ^,^ ^30^. This increase in sympathetic tone appears to correspond with AT, which in this study, corresponded to ≈30% of 1-RM.

### Anaerobic threshold determination by HRV and blood lactate

The determination of AT through indices of HRV was effective and associated with blood lactate responses in patients with CAD who are receiving standard pharmacological therapy. This is an important topic, since these results can be more representative of the CAD population seen clinically. In this context, Machado et al.^25^ assessed HRV indices during progressive upper limb exercise in CAD patients and found medications did not influence the HRV response.

In the present study, the load corresponding to the AT, considering the blood lactate threshold as a parameter during DRET-leg press was obtained at ≈30% of the peak load reached during the 1-RM test (Table 3), which is in accordance with other studies in assessing apparently healthy subjects^14^ ^,^ ^31^.

Figure 2 demonstrates that, although there were agreements among the methods for determining the AT (the mean of the differences was close to zero), the limits of agreement were clinically wide. Other studies have shown the potential use of HRV for the determination of AT/ventilator threshold on a cycle ergometer using rMSSD and SD1 in healthy adults^9^ and in patients with type-2 diabetes^10^. To our knowledge, this is the first study to analyze the behavior of metabolic and autonomic responses during lower limb resistance exercise in CAD patients.

The mean CPX values (peak VO_2_ and predictive VO_2_) indicate that these patients had a well-preserved functional capacity and confirm a maximal effort during the exercise test according to AHA standards^16^. Regarding the response to the DRET, the criteria for interrupting the test was muscle fatigue or excessive rise in SBP (>200 mmHg). All of these patients were included in the data analysis.

### Study perspectives

Our results suggest that HRV may also be considered a useful tool in clinical practice to determine the intensity corresponding to AT. AT was approximately 30% of 1-RM testing for CAD patients with well-preserved functional capacity. HRV analysis using linear and nonlinear methods could be considered an important method for evaluating and understanding cardiac autonomic modulation in CAD patients during dynamic resistance exercise.

In order to establish the correct intensity, it is important to consider that the same exercise may lead to different levels of stress in different patient populations. Several factors, such as body weight, coordination, intention, and perception of the level of effort during resistance exercise, directly interfere with measurements of effort^2^.

## Limitations of this study

The current study has limitations that should be recognized. RMSM and SD2 HRV indices reflect both sympathetic and parasympathetic influences^15^ ^,^ ^29^ and a pure index representative of the sympathetic modulation was not assessed in this study. Moreover, during the exercise protocol with increased load increments every 2 minutes, HRV indices reached steady state in the last minute of each stage of exercise up to AT. However, after AT, this equilibrium condition was not maintained, which is inherent to high exercise intensities. Thus, it is possible that exercise intensities after AT may have affected HRV data capture. Even so, clear trends were apparent in the current investigation. The results found in this study may be protocol-dependent, considering the duration of each load and rest periods between them. The leg press was chosen because it induces more changes in cardiac, ventilatory, and metabolic parameters, but it is necessary to investigate other kinds of resistance exercise. Once the resistance activity stops, the blood pressure decreases quite rapidly so that measuring by auscultation at the end of exercise would do not give a reliable estimation of the blood pressure during exercise. The evaluation of the blood pressure response was limited to the evaluation of discontinuous blood pressure monitoring, measured at the end of the exercise. However, this is still the most widely used method in clinical practice.

## Conclusion

Our results suggest that parasympathetic modulation was reduced during lower extremity resistance exercise, beginning at AT, which occurred at ≈30% of 1-RM. Moreover, HRV may prove to be a feasible tool in clinical practice to determine AT, aiding in setting safe and appropriate exercise intensity parameters in patients with CAD.
